# MR-SP^2^: A Microreactor-Based Workflow for Few-Cell Spatial Proteomics on the Legacy Zeiss PALM MicroBeam

**DOI:** 10.1021/acs.jproteome.5c01231

**Published:** 2026-03-31

**Authors:** Manuel Metzger, Maximilian Maldacker, Tobias Hutzenlaub, Nils Paust, Oliver Schilling, Jan-Niklas Klatt

**Affiliations:** 1 199773 Hahn-Schickard, Georges-Koehler-Allee 103, Freiburg 79110, Germany; 2 Laboratory for MEMS Applications, IMTEK-Department of Microsystems Engineering, 9174 University of Freiburg, Georges-Koehler-Allee 103, Freiburg 79110, Germany; 3 Institute for Surgical Pathology, Faculty of Medicine, Medical Center−University of Freiburg, Breisacher Strasse 115a, Freiburg 79106, Germany; 4 Faculty of Biology, 9174 University of Freiburg, Schaenzlestrasse 1, Freiburg 79104, Germany

**Keywords:** spatial proteomics, sample preparation, LCM, LC-MS/MS, few-cell proteomics, FFPE

## Abstract

Laser capture microdissection (LCM) combined with liquid chromatography–tandem mass spectrometry (LC-MS/MS) enables spatial proteomics at the few-cell level but is constrained by cumulative losses during specimen capture, surface adsorption during processing, and sample transfer prior to LC-MS/MS analysis. The capture-associated losses are particularly relevant for pressure catapulting systems such as the legacy Zeiss PALM MicroBeam, which, despite discontinuation, remains in active use and therefore requires compatible low-loss workflows. We present MR-SP^2^ (microreactor-based sample preparation for spatial proteomics), a one-pot workflow integrating reproducible Zeiss LCM-cut specimen capture, processing with minimized adsorptive losses, and pipetting-free transfer with Evotip disposable precolumns. The workflow was evaluated using a formalin-fixed paraffin-embedded (FFPE) murine kidney tissue analyzed by timsTOF flex LC-MS/MS analysis. Across 50,000 μm^3^ regions (22 cells), MR-SP^2^ modestly improved proteome depth (3381 ± 80 versus 3174 ± 59 proteins). Decreasing sample input further accentuated the advantage of MR-SP^2^ in maintaining higher identification rates, highlighting the successful reduction of the adsorptive losses. At 12,500 μm^3^ (5–6 cells), identifications increased to 1145 ± 188 versus 302 ± 126. At 3125 μm^3^ (1–2 cells), identifications reached 695 ± 112 versus 206 ± 51. MR-SP^2^ improves identification depth for few-cell FFPE samples by nearly 3-fold compared to conventional tube-based workflows.

## Graphical abstract

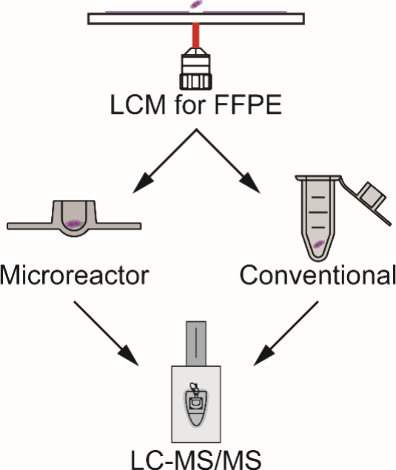

## Introduction

Advances in highly sensitive liquid chromatography–tandem mass spectrometry (LC-MS/MS) proteomics have propelled the study of proteome biology in healthy and diseased tissues.^1,2^ By representing the tissue architecture, spatial proteomics enables precise mapping of protein distribution and abundance, thereby maintaining the spatial context critical for understanding tissue heterogeneity.^3–5^ Currently, mass spectrometry-based methods can identify hundreds to thousands of proteins from increasingly miniaturized samples including single cells.^6^ This capability provides a powerful phenotypic lens to dissect cellular heterogeneity, characterize rare populations, and discover spatially restricted biomarkers, offering fundamental insights for understanding disease mechanisms and developing targeted therapies.^7–9^

To translate these capabilities into practice, spatial proteomic workflows typically combine highly sensitive LC-MS/MS platforms with spatially resolved tissue sampling. In this context, laser capture microdissection (LCM) is the most widely used method for the excision-based miniaturized dissection of tissue specimens for subsequent proteomic analysis. LCM uses a focused laser in combination with a microscope to excise and collect specific microregions from tissues with micrometer precision. Two LCM concepts are prevalent, featuring either gravity-based capture (dropping cut pieces downward) or upward-pressure catapulting.^10^ Importantly, although the catapulting legacy Zeiss PALM MicroBeam (Zeiss LCM) predates many recent spatial proteomics workflows, it remains a prevalent legacy device with a broad installed user base (see Figure S1) and is therefore still a common device for spatially defined tissue sampling. In subsequent downstream processing for mass spectrometry-based proteomics and analysis, the achievable proteomic depth depends on the LC-MS/MS instrumentation and the prevention of sample loss during the sample preparation, which consists of LCM-cut specimen capture, sample processing (protein extraction, reductive alkylation, digestion, and acidification), and transfer to the LC-MS/MS system. Because instrument sensitivity and acquisition time are often constrained by established laboratory configurations, improving sample recovery across LCM capture, low-input processing, and LC interfacing remains a practical and broadly applicable route to increasing proteomic depth.

Losses during sample preparation for spatial proteomics can arise from several sources. One major limitation is the partial or complete loss of LCM-cut specimens during transfer into and within the reaction vessel, a problem that is especially pronounced for Zeiss LCM.^10,11^ In addition, adsorptive losses of proteins and peptides to the inner surfaces of reaction vessels occur during processing steps such as extraction, reductive alkylation, and digestion.^12^ A further source of loss arises from pipetting steps required for transferring samples to the LC-MS/MS system. Especially in few-cell setups, a single pipetting step can lead to a 50% decrease in proteomic identifications.^13^

Overcoming these limitations is critical to fully harness the potential of spatial proteomics for widespread research and clinical applications.^14–16^ Recent approaches have increasingly relied on automated liquid handling for low-input sample processing, which has helped in reducing surface exposure and transfer losses.^3,17,18^ Yet, these approaches typically depend either on liquid-handling platforms for multiwell plate processing with gravity-based Leica LMD^3,18^ or liquid-handling platforms for specific chip collection formats for catapulting Zeiss LCM (e.g., nanoPOTS^17^) to avoid adsorptive losses of the sample.^19^ Even though these approaches enable sample preparation with minimal adsorptive losses, reproducible handling of open, nanoliter volumes requires complex and costly instrumentation, such as dispensing systems,^3,17^ and can be prone to evaporation during incubation steps, which can create variance in digestion efficiency.^20^ As evaporation is temperature-dependent, temperature limits can be imposed on open systems^3,17,21^ impairing protein extraction from FFPE samples.^22^ As a result, conventional tube-based workflows remain widely used despite using larger volumes and multiple transfer steps, such as LCM-cut specimen transport within reaction vessels and pipetting into the LC-MS/MS interface.^23,24^ Because many current low-loss approaches rely on specialized platforms (like liquid-handling platforms), they may not translate directly to many laboratories operating the legacy Zeiss LCM with conventional tube-based handling, which represents a substantial segment of the potential spatial proteomic user base. Accordingly, cumulative losses associated with Zeiss LCM during specimen capture, processing, and downstream interfacing with established LC-MS/MS systems (e.g., combination of Evosep One and timsTOF flex) remain insufficiently addressed, underscoring the need for a low-loss sample preparation strategy compatible with the constraints set by Zeiss LCM.

We address this gap with a microreactor-based sample preparation workflow for spatial proteomics (MR-SP^2^). MR-SP^2^ centers on a novel microreactor that is compatible with Zeiss LCM for reproducible capture of LCM-cut specimens. Sample processing within the microreactor is conducted in a closed, one-pot system, effectively minimizing evaporation and reducing adsorptive losses. Sample transfer to LC-MS/MS is performed by clipping the microreactor on an Evotip, hence avoiding pipetting of the sample and eliminating the associated adsorptive losses. MR-SP^2^ provides a replicable low-input spatial proteomic workflow suitable for legacy Zeiss LCM and established LC-MS/MS (e.g., combination of Evosep One + timsTOF flex) instrumentation.

## Experimental Section

### Animals and Ethics

A murine kidney tissue was obtained from C57BL/6 mice originally bred for maintenance of the colony. The animals were housed under specific pathogen-free (SPF) conditions in accordance with the Federation of Laboratory Animal Science Associations (FELASA) guidelines.^25,26^ Sacrifice and organ retrieval were performed for scientific purposes in strict compliance with the German Animal Welfare Act (Tierschutzgesetz §4 Abs. 3). The procedure was notified to and approved by the local Animal Welfare Officer (Tierschutzbeauftragter) and the responsible authorities (Regierungspräsidium Freiburg).

### LCM System Survey

We performed a targeted literature screening to quantify reporting of LCM manufacturers in spatial proteomics and related workflows. PubMed was queried for laser microdissection-related articles, and the resulting PubMed identifiers were mapped to PubMed Central IDs via the Entrez linking interface. For entries with full text in PubMed Central, XML articles were downloaded, and Methods/Materials and Methods sections were extracted. These sections were searched for predefined vendor-specific keyword patterns corresponding to Zeiss PALM, Leica LMD, MMI CellCut, and Thermo/Arcturus systems (Table S1), and each article was assigned to one of these vendor categories or to “Other/Unspecified” when no specific instrument could be identified.

### Initial Tissue Processing

Following dissection, the kidney was fixed in 4% formalin (Sigma-Aldrich, St. Louis, USA) overnight. After fixation, the tissue was dehydrated through a graded ethanol series, cleared in a series of xylene (Carl Roth, Karlsruhe, Germany) washing steps, and infiltrated with molten paraffin wax. The paraffin-impregnated tissue was embedded in a paraffin block and stored at room temperature until further use. The tissue was then cut into 5 μm-thick sections and mounted on PEN slides (Carl Zeiss Microscopy, Jena, Germany) for laser microdissection. Before conducting LCM, the section was deparaffinized in consecutive steps of 99%, 96% 70% xylol, and 50% ethanol (Carl Roth) and a final wash procedure in 70% ethanol (Carl Roth) before being rehydrated and kept in water until dissection.

### Manufacturing of the Microreactor

The microreactor (Figure 1A) was manufactured using thermoforming.^27,28^ For this, the design was created using SolidWorks 2021 (Dassault Systèmes, Vélizy-Villacoublay, France) and subsequently milled in poly­(methyl methacrylate) (PMMA) using a Kern Micro HD (Kern Mikrotechnik, Hohenlohe, Germany) milling machine. The PMMA master was molded using Elastosil RT607 polydimethylsiloxane (PDMS, Wacker Chemie, München, Germany). Thermoforming was performed on a hot embossing machine (Wickert Maschinenbau, Landau in der Pfalz, Germany) using the PDMS mold and 2× 400 μm COC foil (TekniPlex, Wayne, USA) consisting of a low-melting side (COC 8007) and a high-melting side (COC 6013). After thermoforming, the microreactor was cut out by using a laser cutter (Universal Laser Systems, Scottsdale, USA). No surface passivation was applied to the microreactor’s surface.

**Figure 1:**
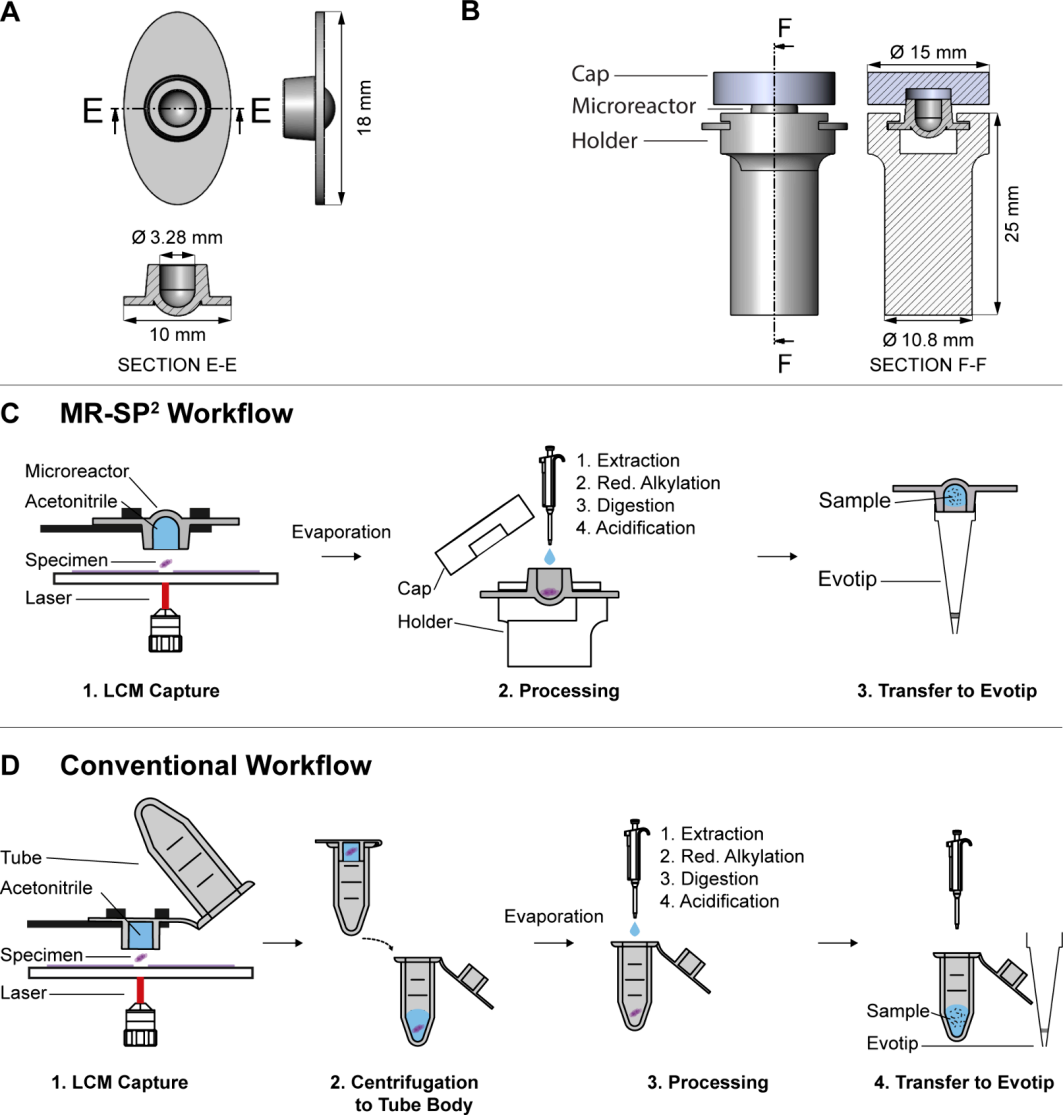
(A) Technical drawing of the microreactor; all dimensions in mm. (B) All three components assembled together with the microreactor in the holder and the cap attached to avoid evaporation; all dimensions in mm. (C) MR-SP^2^ workflow: (1) The microreactor filled with 20 μL of acetonitrile captures a Zeiss LCM-cut specimen and is subsequently mounted in a 3D-printed holder, and the acetonitrile is evaporated in a SpeedVac under rotation. (2) After evaporation, a closed one-pot workflow (extraction, reductive alkylation, digestion, and acidification) is performed, with a polymer cap applied after each step to prevent evaporation. (3) The microreactor is clipped onto an Evotip, enabling pipetting-free transfer to LC–MS/MS. (D) Reference tube-based workflow: (1) A tube cap filled with 40 μL of acetonitrile captures an LCM-cut specimen by Zeiss LCM. (2) The specimen and acetonitrile are centrifuged to the tube body, and acetonitrile is evaporated in a SpeedVac under rotation. (3) After evaporation, a closed one-pot workflow (extraction, alkylation, digestion, and acidification) is performed, with the tube cap applied after each step to prevent evaporation. (4) The sample is pipetted into an Evotip for LC-MS/MS analysis.

For manufacturing of the microreactor cap (Figure 1B), a 3D printing process using the Arburg Freeformer 300-3X (Arburg, Lossburg, Germany) was employed using APEL COC 6013T (Mitsui Chemicals, Minato, Japan) as a material, and Armat 11 (Arburg) served as a support material during the printing process. A cylinder with a diameter of 15 mm and a thickness of 4 mm was printed. Alternatively, a commercially available 4 mm polymer sheet can be used for further processing. After printing, a hole with a depth of 2 mm was drilled into the cylinder using a 5.6 mm drill bit. To prevent thermobonding of the microreactor and the cap of the microreactor during the 95 °C extraction step, the drill hole was coated with a 10% w/w solution of PTFE (DuPont Fluoroproducts, Wilmington, USA) in Fluorinert FC-40 ^29^ (Sigma-Aldrich) and the Fluorinert was subsequently evaporated. The cap may be replaced with a sealing foil for PCR applications to prevent evaporation. As a convenient handling tool without an impact on workflow performance, the holder for the microreactor (Figure 1B) was also 3D printed using the same printing process as for the cap. CAD models for the cap and holder can be found in the Supporting Information.

### Laser Capture Microdissection

Laser capture microdissection was conducted using the Zeiss PALM MicroBeam platform connected with a Zeiss microscope (MBC01092), ControlBox (2_Rev. 4.2), a stage (Stage IIB), and the capture device (RoboMover) mounted with the Collector Set SingleTube II 500. The specimens were cut with a CryLaS UV-Laser and controlled with the software installations of PALMRobo (V 4.6.0.4), MTB (V 1.8.1.5), AxioVision (4.8.3.0), and ControlBox (1,170,146M). The tissue was dried before the tube or microreactor. To prevent biological intratissue variance overshadowing the technical variance, we selected homogeneous renal cortical tissue sections (Figure S2A,B). The tissue was dissected in “Cut” mode for the given tissue area with different sizes before transferring the LCM-cut specimen to the reaction vial with a series of LPC shots (Figure S2C–E).

For conventional tube capture, 200 μL PCR tubes were used (PCR microcentrifuge tube PP, 0.2 mL, 8-strips, Nerbe Plus, Winsen, Germany). For capture, the caps of the tubes were filled with 40 μL of acetonitrile. The successful transfer in the acetonitrile volume was confirmed visually before sample processing. A subsequent transfer step of the acetonitrile and LCM-cut specimen from tube cap to tube body was performed by short centrifugation using a minicentrifuge with a maximum of 2000*g*.

For microreactor capture, due to lower cavity volume, the microreactor was filled with 20 μL of acetonitrile. The microreactor was placed in the position taken by the cap for the conventional tube capture.

In both cases, the acetonitrile was evaporated by centrifugal vacuum evaporation after capture using a Speedvac (Eppendorf, Hamburg, Germany).

### Sample Processing for LC-MS/MS

An adapted version of the one-pot workflow for few-cell proteomics by Tsai et al.^30^ was performed. For extraction, 5.0 μL of 0.2% dodecyl-β-d-maltoside (DDM, Carl Roth) in 5.0 mM tris­(2-carboxyethyl)­phosphine hydrochloride (TCEP, Sigma-Aldrich) was added with incubation at 95 °C for 1 h. Reductive alkylation follows the extraction with the addition of 1.0 μL of 60.0 mM chloroacetamide (CAA, Sigma-Aldrich) in 5.0 mM TCEP (final CAA concentration of 10 mM) at 37 °C for 30 min. Then, 1.0 μL of 175 mM ammonium bicarbonate (Sigma-Aldrich) in 5 mM TCEP was added for pH stabilization, directly followed by 1.0 μL of a trypsin (50 ng/μL, Serva) and LysC (25 ng/μL, Serva, Heidelberg, Germany) solution for enzymatic digestion. Enzymatic digestion was carried out for 16 h at 37 °C. Samples were then acidified by the addition of 12 μL of 10% formic acid (FA, Sigma-Aldrich).

### LC-MS/MS

Evotips were preconditioned according to manufacturer guidelines. Samples were transferred to Evotips (Evosep, Odense, Denmark) using either manual pipetting (for conventional tube capture) or by centrifugal transfer (microreactor clipped on Evotips, see Figure S3) and centrifuged for 1 min at 800*g*. The samples were loaded with a volume of 20 μL and washed twice with 20 μL of 0.1% FA in H_2_O. The samples were kept in 0.1% FA in H_2_O at 4 °C until MS acquisition. Peptides were analyzed with a timsTOF flex (Bruker Daltonics, Billerica, USA) coupled to an Evosep One equipped with a 15 cm performance column (EV1137) tempered to 40 °C. Peptide separation was conducted with 0.1% FA in MS-grade water and 0.1% FA in acetonitrile in a 30 SPD gradient and injected utilizing the captive spray source with a 20 μm ZDV sprayer (Bruker) and a capillary voltage of 1600 V.

The acquisition was conducted with one MS1 followed by 16 diaPASEF scans over two ion mobility windows per scan within the ion mobility range of 0.6–1.6 1/K0 and *m*/*z* range of 400–1200 Th by 25 Th windows (Table S2). The accumulation and ramp time were set to 150 ms. Precursors within the ion mobility range of 0.6–1.6 1/K0 and within the *m*/*z* range of 400–1200 Th were selected for fragmentation with one MS1 cycle, followed by 32 MS2 scans from two PASEF cycles each. Precursors were fragmented with a collision energy of 20 eV at 1/K0 = 0.6 s cm^–2^ and 59 eV at 1/K0 = 1.6 s cm^–2^ increasing linearly between those values. MS2 spectra were acquired in the range of 100–1700 Th.

### Data Analysis

LC-MS/MS data were searched with DIA-NN 2.2.0 ^31,32^ against the *Mus musculus* proteome database (EBI UniProt Reference Proteome UP000000589, downloaded October 2022; one representative protein per gene, 21,997 entries) with appended iRT peptides and common contaminants. A spectral library was predicted from the given fasta file utilizing tryptic peptides and allowing for a maximum of 1 missed cleavage. Precursors with a peptide length between 7 and 50 Th within an MS1 range of 400–1200 Th were matched with fragment ions within an MS2 range of 100–1700 Th. N-terminal M excision and oxidation at M were set to variable modifications, and carbamidomethylation at C was set as fixed modification. Protein inference was activated, scoring was run in generic mode, and NNs were selected for learning with QuantUMS^33^ and conducted in high precision mode. Only identifications with a *q*-value <0.01 were retained for analysis. Identification numbers were reported from two separate DIA-NN analyses without and with applying MBR, with MBR being confined to LC-MS/MS analyses of equal input amounts for the respective condition. Protein abundances were determined using DIA-NN’s integrated MaxLFQ normalization, and peptide quantification was based on the Precursor.Quantity metric. Data analysis was done using an in-house Python script including packages shown in the Supporting Information (see Data Availability). Statistical significance was evaluated with a Mann–Whitney *U* test for all pairwise comparisons of peptide and protein identifications and for the comparison of average peptide lengths where a Student’s *t* test was used.

## Results and Discussion

### Continued Usage of the Legacy Zeiss PALM LCM System

A survey of LCM-based proteomic publications in the past decade (Figure S1 and Table S1) revealed that the Zeiss PALM system remains the second most widely used LCM platform. This substantial share of active usage is notable, given that the Zeiss PALM has been discontinued but the pressure catapulting mechanism of the Zeiss PALM represents a still-prevalent modality in the field. The persistent use of this system across laboratories, combined with the lack of optimized low-input workflows, motivated the development of MR-SP^2^ to address the commonly encountered shortcomings in specimen loss during capture, surface adsorption during processing, and transfer inefficiencies to the LC-MS/MS system, which are inherent to conventional tube-based implementations on this platform.

### The MR-SP^2^ Workflow Mitigates Sample Loss in Zeiss LCM

The microreactor (Figure 1A,B) is the core component of the MR-SP^2^ workflow (Figure 1C), designed to address the limitations in the LCM-cut specimen capture, sample processing, and transfer to LC-MS/MS.

The microreactor is fully compatible with the Zeiss PALM MicroBeam platform and its RoboMover for upward-pressure catapulting LCM. To avoid specimen loss due to adherence to capture adhesives,^34^ fluid-assisted collection^17^ with acetonitrile is employed. The microreactor requires only 20 μL of acetonitrile for specimen capture, simplifying localization and visual verification compared to the higher volume of the conventional workflow (40 μL capture volume, Figure 1D).

By capturing LCM-cut specimens directly in the microreactor, postdissection tissue transport is eliminated for the MR-SP^2^ workflow. Conventional workflows, however, require an additional centrifugation step to transfer specimens from the tube cap to the tube body, which resulted in specimen loss even after confirmed initial capture (Figure S4, 22 cells-conventional-R6). To conclude the capture, the specimen is localized at the base of the reaction vessel by solvent evaporation under rotation. In theory, the microreactor may be compatible with other LCM systems (e.g., Leica LMD) given that a suitable tube holder is available since the outer geometry follows a conventional tube cap form factor. However, compatibility was not evaluated here and remains beyond the scope of the present study.

For sample processing, the microreactor’s cavity volume is optimized to a total workflow volume of 20 μL, minimizing the area exposed to the inner surface of the reaction vessel. It can withstand temperatures above 95 °C, enabling high-temperature protein extraction of FFPE tissues. To prevent evaporation, the microreactor is sealed with a cap (Figure 1B) before the incubation steps.

To enable pipetting-free sample transfer to LC-MS/MS, the microreactor is designed to clip onto an Evotip, significantly reducing the adsorptive losses associated with pipetting steps. Both the MR-SP^2^ and conventional workflows use a total processing volume of 20 μL (see the Experimental Section). By minimizing the exposed surface area and eliminating pipetting during sample transfer, MR-SP^2^ reduces the cumulative surface exposure by 64% compared to the conventional workflow (33.4 mm^2^ vs 92.8 mm^2^, Figure S5), thereby substantially decreasing nonspecific protein and peptide binding.

### MR-SP^2^ Yields Improved Proteome Depth Compared to Conventional Tube-Based Zeiss PALM Sample Processing

We evaluated capture efficiency for conventional tubes and the microreactor by LCM of 100 × 100 μm^2^ regions from 5 μm murine kidney sections in 16 replicates each. Visual inspection confirmed successful specimen capture in 15 of 16 microreactor-captured samples (94%), whereas only 12 of 16 samples (75%) were successfully captured using the conventional tube-based workflow. In addition, the 2-fold reduction in capture volume in the microreactor facilitated visual confirmation of specimen presence, enabling more reliable verification compared to the tube-based workflow.

LCM-cut specimens were further processed and analyzed by LC-MS/MS to examine whether the MR-SP^2^ workflow approach translates into deeper proteomic depth (Figure 2A,B). We are reporting identification numbers without and with applying MBR, with MBR being confined to LC-MS/MS analyses of equal input amounts for MR-SP^2^ and conventional. Notably, one conventionally processed sample yielded markedly low identifications (515 peptides and 219 proteins) despite confirmed dissection and transfer into the tube cap (Figure S4). By facilitating visual confirmation of the specimen presence and eliminating subsequent transfer steps within the microreactor, the MR-SP^2^ workflow features an increased overall procedural robustness. In order to evaluate the workflow performance, the low identification sample of the conventional workflow was not included in the further analysis.

**Figure 2:**
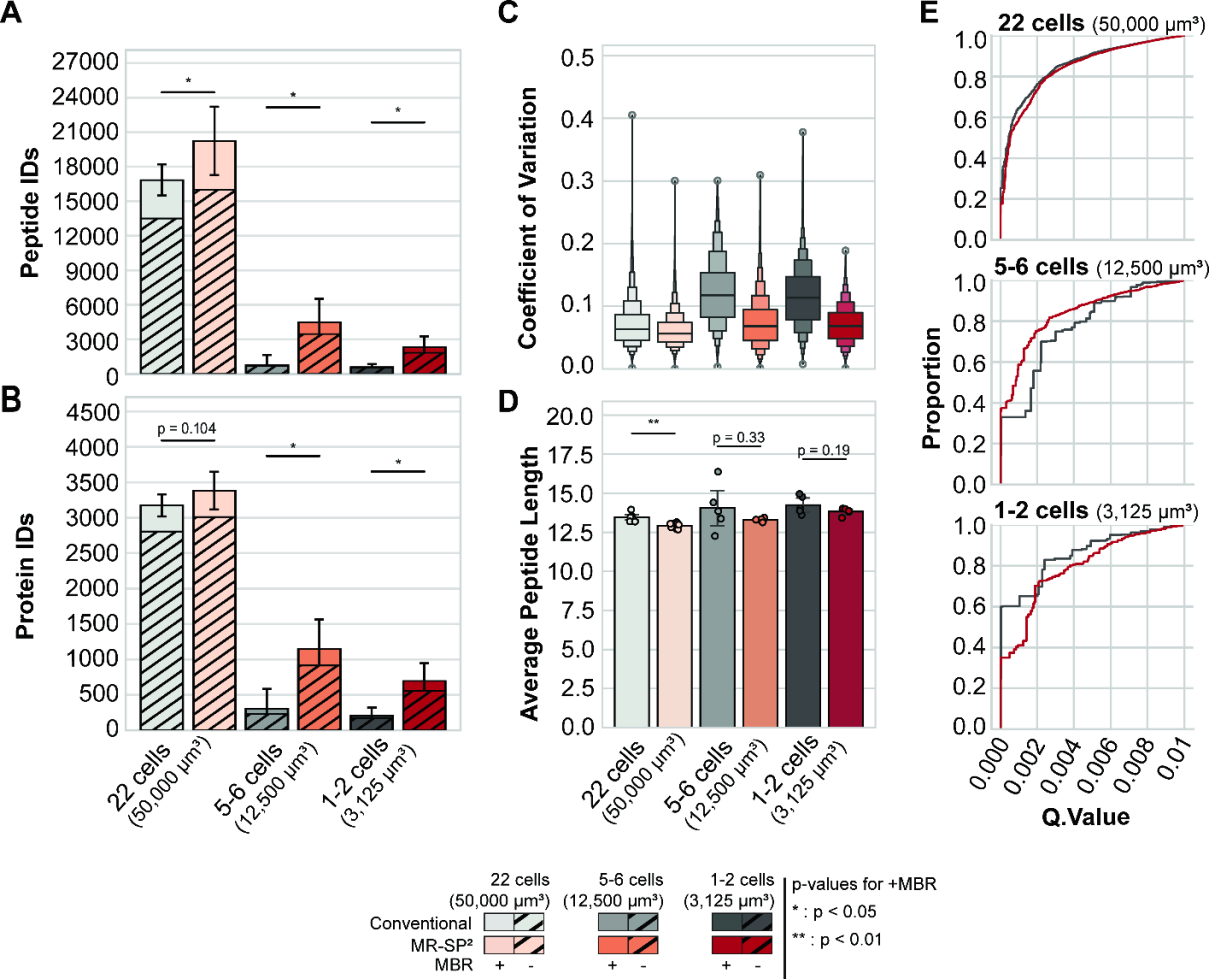
(A) Number of identified peptides and (B) proteins from 22 (50,000 μm^3^), 5–6 (12,500 μm^3^), and 1–2 cells (3,125 μm^3^) of an FFPE murine kidney slide. Bars with patterns denote identifications without applying the match between runs algorithm (MBR), while bars without patterns indicate additional matches assigned by MBR within the same condition. Error bars represent the standard error of the mean for *n* = 7, *n* = 11, and *n* = 5 samples for 22 cells (50,000 μm^3^)-conventional, 22 cells (50,000 μm^3^)-MR-SP^2^, and the remaining conditions, respectively. Significance was tested using a Mann–Whitney *U* test on identification with MBR. (C) Coefficient of variation for the log_2_ transformed precursor intensity among peptides identified in at least 3 precursors per condition. (D) Peptide length in amino acids. Significance was tested with Student’s *t* test. (E) Q.Value distribution for different input amounts in cell equivalents for MR-SP^2^ and conventional sample preparation.

MR-SP^2^ demonstrated superior proteomic depth across different input amounts (Figure 2A,B). For a dissected volume of 50,000 μm^3^ (22 cells^35,36^ ), it yielded an average of 16,021 ± 1009 peptide identifications (20,229 ± 895 with MBR; *n* = 11), a significant increase over the 13,514 ± 740 (16,852 ± 513 with MBR; *n* = 7) identified peptides from the conventional workflow (Figure 2A; *p* < 0.05, Mann–Whitney *U* test). The improved depth was less pronounced on the protein level with an increase of approximately 6% more proteins (Figure 2B; *p* = 0.10, Mann–Whitney *U* test), indicative of improved sequence coverage of individual proteins. We further probed reduced input amounts in addition to the 50,000 μm^3^ LCM-cut specimens: 12,500 μm^3^ (5–6 cells^35,36^ ) and 3125 μm^3^ (1–2 cells^35,36^ ). For an input of 5–6 tissue cells (12,500 μm^3^), the MR-SP^2^ (*n* = 5) yielded an average of 3467 ± 902 peptide identifications (4495 ± 912 with MBR), corresponding to a >5-fold increase as compared to conventional workflow (*n* = 5), which yielded 685 ± 420 peptide identifications (801 ± 380 with MBR) (*p* < 0.05, Mann–Whitney *U* test). Unlike for the 22-cell cell input (50,000 μm^3^), the improved peptide depth also translates to a strongly improved protein depth: the MR-SP^2^ workflow resulted in an average of 914 ± 216 (1145 ± 188 with MBR) protein identifications, which is >3-fold more than the 231 ± 135 (302 ± 126 with MBR) proteins identified by the conventional workflow (*p* < 0.05, Mann–Whitney *U* test). For an input of 1–2 tissue cells (3125 μm^3^), the microreactor workflow (*n* = 5) yielded an average of 1841 ± 480 peptide identifications (2320 ± 423 with MBR), corresponding to a >3-fold increase as compared to the conventional workflow (*n* = 5), which yielded 541 ± 170 peptide identifications (601 ± 111 with MBR) (*p* < 0.05, Mann–Whitney *U* test). The improved peptide depth also translates to improved protein depth: the MR-SP^2^ workflow resulted in an average of 557 ± 135 (695 ± 112 with MBR) protein identifications, which is >3-fold more than the 176 ± 73 (206 ± 51 with MBR) proteins identified by the conventional workflow (*p* < 0.05, Mann–Whitney *U* test).

To assess whether LC carry-over affected protein and peptide identifications, we analyzed empty Evotips processed and measured under identical LC–MS/MS conditions (Figure S6). Empty runs showed no detectable signal in the total ion chromatograms (Figure S6A–C) and yielded only negligible numbers of identified precursors and MS1 intensities compared to experimental acquisitions (Figure S6D,E). In contrast, experimental runs exhibited a pronounced increase in both precursor identifications and the cumulative MS1 signal across all cut sizes. These results demonstrate that the observed peptide and protein identifications are clearly distinguishable from the background and are not attributable to carry-over effects.

Previous observations already reported that an increased surface area causes significant sample losses due to adsorption with decreasing sample inputs being affected disproportionately.^37^ In line with this, we observed a significant increase in peptide and protein identifications, especially for reduced input levels, and linked this to the omission of one pipetting step in MR-SP^2^.

To determine the coefficient of variation (CV) of the precursor intensities per processing, we selected precursors with at least three valid values per condition. At 22 cells (50,000 μm^3^, sampled from comparable kidney areas), both workflows indicated a high quantitative accuracy with mean CVs of 0.070 ± 0.036 from 21,018 and 0.061 ± 0.027 from 29,246 precursors for the conventional and MR-SP^2^ workflow. Shared precursors also showed high accuracy with shared CV values of 0.069 ± 0.034 and 0.061 ± 0.026 for the conventional workflow and MR-SP^2^, respectively. In addition, the majority of all precursors yielded a CV < 0.15 (Figure 2C). A multilaboratory assessment of reproducibility, qualitative, and quantitative performance of LC-MS/MS based proteomics suggested bioanalytical acceptance of <20% CV with our CV values comparing favorably.^38^ Correlation analysis of precursor intensities between technical replicates (Figure S7) revealed high intracondition *R* values, indicating good reproducibility despite tissue heterogeneity from distinct yet homogeneous regions dissected by LCM. Notably, MR-SP^2^ showed substantially higher intracondition correlations than the conventional workflow at 5–6 and 1–2 cell inputs.

Quantitative accuracy remained stable, even under extremely low input. At 5–6 cells (12,500 μm^3^), variability fell from 0.12 ± 0.06 based on 462 precursors to 0.07 ± 0.04 based on 5171 precursors (for shared precursors; *n* = 388, 0.129 ± 0.055 vs 0.106 ± 0.054 for conventional vs MR-SP^2^, respectively). At 1–2 cells (3,125 μm^3^), it decreased from 0.116 ± 0.051 on 566 precursors to 0.071 ± 0.031 on 2774 precursors (Figure 2C; for shared precursors; *n* = 506, 0.115 ± 0.051 vs 0.075 ± 0.034 for conventional vs MR-SP^2^, respectively). This substantial reduction in variance highlights the robustness of MR-SP^2^ and its suitability for a highly accurate analysis of few-cell proteomes. The data suggest that by the elimination of one transfer step and the reduction of the exposed total surface, entropic peptide-level losses can be avoided that would otherwise affect reproducible quantification across several LC-MS/MS runs.

In addition, we determined the average length of the identified peptides per LC-MS/MS run (Figure 2D). For the input amount of 22 cells (50,000 μm^3^), MR-SP^2^ derived peptides showed a minor (less than one residue on average), albeit significant tendency for reduced amino acid length (12.9 ± 0.05 versus 13.5 ± 0.09; *p* < 0.01, Student’s *t* test). A similar, yet not significant trend was observed for the reduced input amount of 5–6 cells (12,500 μm^3^) with 13.3 ± 0.05 and 14.1 ± 0.7 amino acids in length for MR-SP^2^ and the conventional workflow, respectively (*p* = 0.33, Student’s *t* test). Similarly, LC-MS/MS runs from 1 to 2 cells (3125 μm^3^) exhibited a similar average peptide length with 13.8 ± 0.1 and 14.3 ± 0.3 amino acids in length. Overall, MR-SP^2^ peptides show only a marginal reduction in average length compared with the conventional workflow. Importantly, this minor difference does not produce a consistent shift across LC-MS/MS runs, indicating that peptide size distributions are largely maintained even at minimal input levels.

Furthermore, we employed the Q.Value metrics of DIA-NN as a parameter for spectral quality of sequence identifications (Figure 2E). No major differences between the conventional and the MR-SP^2^ workflow are evident. This suggests that the increased identifications observed in the MR-SP^2^ workflow are not driven by lowered confidence thresholds or increased false positives but rather reflect true improvements in recovery.

### Gains in MR-SP^2^ Originate from Low Abundant Peptides

We assessed to which extent each workflow is contributing to the identification of specific peptide subsets. To this end, we used the accumulated peptide identifications per workflow, including peptides identified in a single LC-MS/MS run with a global *q*-value <0.01. For all three input amounts, there was a sizable proportion of peptides uniquely identified in either the conventional or the MR-SP^2^ workflow (Figure 3A–C).

**Figure 3:**
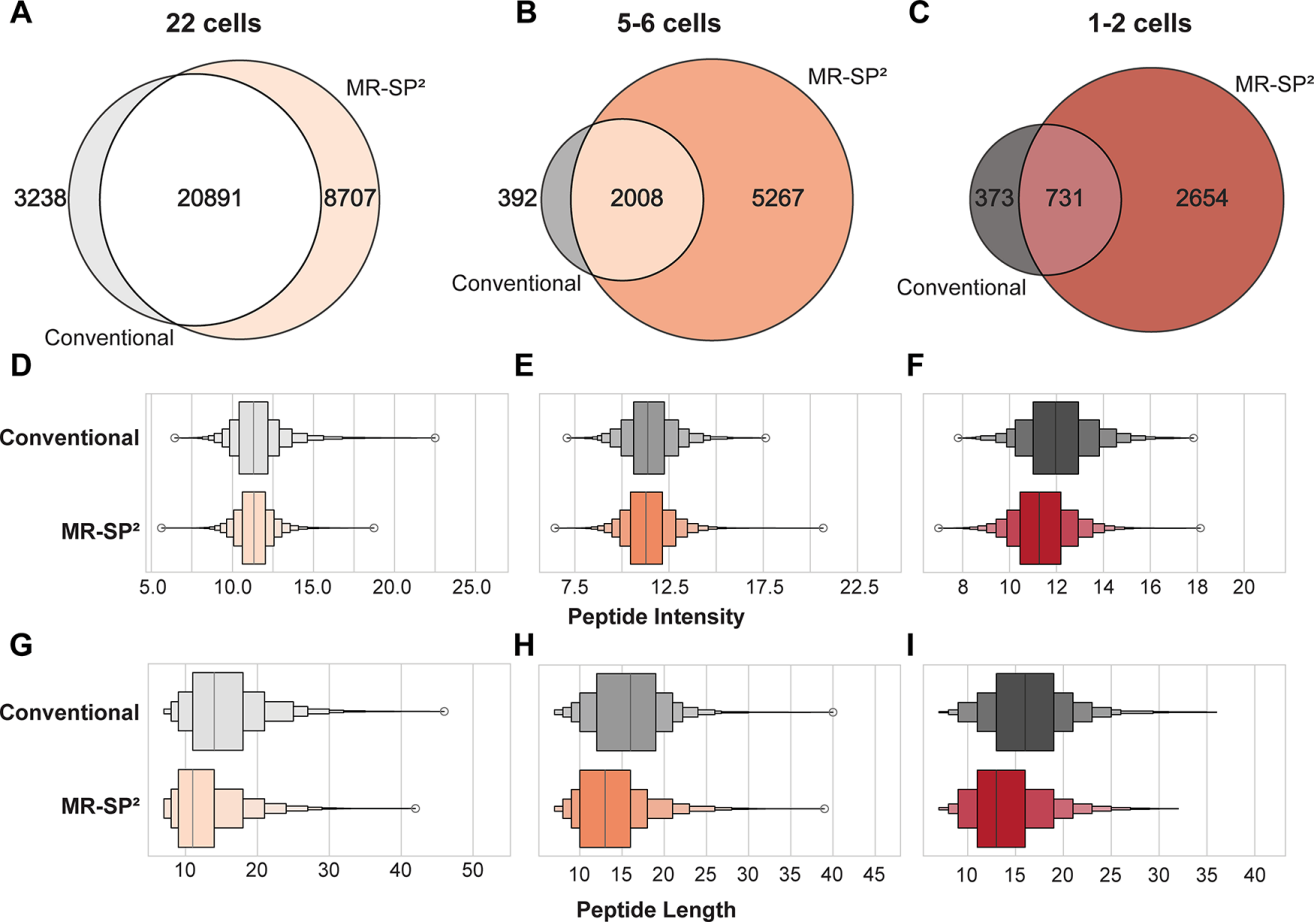
Cumulative Venn diagram of all identified peptides for MR-SP^2^-based sample preparation or conventional sample preparation resulting from 22 (50,000 μm^3^) (A), 5–6 (12,500 μm^3^) (B), or 1–2 (3125 μm^3^) (C) cells from FFPE murine kidney being processed. MBR was activated across all samples from the same condition. Log_2_ peptide intensity of unique peptides from A–C for either the MR-SP^2^ or conventional workflow from 22 (50,000 μm^3^) (D), 5–6 (12,500 μm^3^) (E), or 1–2 (3,125 μm^3^) (F) cell equivalents being processed. Peptide length of unique peptides for either the MR-SP^2^ or conventional workflow from 22 (50,000 μm^3^) (G), 5–6 (12,500 μm^3^) (H), or 1–2 (3125 μm^3^) (I) cells being processed. Identification, peptide intensity, and peptide length from *n* = 7, *n* = 11, and *n* = 5 samples for 22 cells (50,000 μm^3^)-conventional, 22 cells (50,000 μm^3^)-MR-SP^2^, and the remaining conditions, respectively.

For unique peptides from MR-SP^2^-processed samples, we obtained consistently lower MS2-level intensities compared to peptides unique to the conventional workflow, a trend that became most pronounced at the lowest input amounts (Figure 3D–F). At 22 cells (50,000 μm^3^), the mean log_2_ intensity was 11.3 ± 1.2 for MR-SP^2^ and 11.4 ± 1.6 for the conventional workflow. At 5–6 cells (12,500 μm^3^), intensities were 11.4 ± 1.3 versus 11.5 ± 1.4, and at the 1–2 cell level (3125 μm^3^), 11.4 ± 1.3 versus 12.0 ± 1.6. Thus, the data showcase the conceptual reduction of the exposed surface area in MR-SP^2^ is enabling the detection of low abundant unique peptides that would otherwise be below the limit of detection and are prone to adsorptive losses.^39,40^

We also determined the observable peptide sequence length (Figure 3G–I). At 22 cells (50,000 μm^3^), MR-SP2 peptides were shorter on average (11.3 ± 1.2 amino acids) than those from the conventional workflow (14.8 ± 5.5). This difference persisted at 5–6 cells (12,500 μm^3^; 13.4 ± 4.4 vs 15.7 ± 4.7) and was also apparent at 1–2 cells (3125 μm^3^; 13.8 ± 4.2 vs 16.0 ± 4.6). The decrease in peptide length as an indicator for better digestion efficiency^41^ hereby demonstrates that the MR-SP^2^ contributes to enhanced cross LC-MS/MS run quantification of low abundant proteins inferred from low abundant peptides.

Collectively, these results show that differences in intensity and peptide length become more prominent with lower sample input. The consistent trend toward shorter and less intense peptides in the MR-SP^2^ workflow indicates improved transfer of low abundant peptides from the sample to the instrument due to reduced absorption and transfer-induced sample loss.

## Conclusions

While established approaches have increased proteomic depth in spatial proteomics, the discontinuation of the Zeiss PALM LCM system has left this pressure catapulting configuration without further dedicated workflow development despite its continued widespread use. Conventional tube-based implementations for Zeiss PALM remain accessible but are associated with specimen losses during LCM capture, adsorption to processing surfaces, and transfer losses prior to LC–MS/MS analysis, which collectively constrain the achievable depth. Further gains in identification numbers could, in principle, be achieved through optimization of LC–MS/MS acquisition parameters; however, this falls outside the scope of the present study.

MR-SP^2^ addresses losses arising from LCM capture, low-input processing, and LC interfacing through its microreactor design. By enabling transfer-free and pipetting-free one-pot processing, MR-SP^2^ showed a reduction in specimen loss and exposed surface area by 64% compared to a conventional tube-based workflow, leading to more than three times higher peptide and protein identifications from low FFPE input down to 3125 μm^3^ (1–2 cells). At the same time, MR-SP^2^ maintains quantitative robustness across all tested tissue amounts with mean CVs below 0.10, supporting proteomic characterization of spatially defined tissue microregions. The workflow design and compatibility with broadly installed Zeiss LCM and established LC-MS/MS instrumentation may facilitate the easy adoption in other laboratories.

## Supplementary Material









## Data Availability

Raw mass spectrometry data associated with the diaPASEF acquisition method have been deposited in MassIVE under accession MSV000099955. Analysis scripts are accessible through a GitHub link: https://github.com/SchillingLabProteomics/MR-SP-A-Microreactor-Based-Workflow-for-Few-Cell-Spatial-Proteomics-.git.

## Notes

(Figure S1) Distribution of different LCM platforms across publications from the past decade, (Figure S2) scan of the processed murine kidney before and after LCM, (Figure S3) physical components used to perform the MR-SP^2^ workflow, (Figure S4) individual LC-MS/MS runs showing peptide and protein identifications, (Figure S5) CAD model showing the surface area for MR-SP^2^ and conventional workflow, (Figure S6) LC-MS/MS runs of empty Evotips, (Figure S7) heatmap of Pearson’s correlation across all samples, (Table S1) vendor-specific keywords used in the literature survey, and (Table S2) diaPASEF acquisition scheme (PDF)
Supplemental video showing the workflow steps to perform MR-SP^2^ (MOV)
STEP file containing the holder for the microreactor for 3D printing (ZIP)
STEP file containing the cap for the microreactor for replication (ZIP)

## Abbreviations

CAA: chloroacetamide
COC: cyclic olefin copolymer
CV: coefficient of variation
DDM: dodecyl-β-d-maltoside
FA: formic acid
FFPE: formalin-fixed paraffin-embedded
LC-MS/MS: liquid chromatography–tandem mass spectrometry
LCM: laser capture microdissection
MBR: match between runs
MR-SP^2^: microreactor-based sample preparation for spatial proteomics
PMMA: poly­(methyl methacrylate)
PDMS: polydimethylsiloxane
TCEP: tris­(2-carboxyethyl)­phosphine hydrochloride
