# Supplementary material for: MR-SP2: A Microreactor-Based Workflow for Few-Cell Spatial Proteomics on the Legacy Zeiss PALM MicroBeam
Source: J Proteome Res. 2026 Mar 31;25(5):2486–94. doi: 10.1021/acs.jproteome.5c01231 (PMC13140122; doi:10.1021/acs.jproteome.5c01231)
Supplement: Supplementary file 1 [file pr5c01231_si_001.pdf]

Supplemental Information

MR-SP²: Unlocking Few-Cell Spatial Proteomics for the Zeiss PALM Laser Microdissection Platform

Manuel Metzger [1,2,†], Maximilian Maldacker [3,4,†], Tobias Hutzenlaub [1,2], Nils Paust [1,2], Oliver Schilling [3,] and Niklas Klatt [1,2,*]*

†, Contributed equally*

** Corresponding Authors; Niklas.Klatt@Hahn-Schickard.de and Oliver.Schilling@mol-med.uni-freiburg.de*

[1] Hahn-Schickard, Georges-Koehler-Allee 103, 79110 Freiburg, Germany

[2] Laboratory for MEMS Applications, IMTEK-Department of Microsystems Engineering, University of Freiburg, Georges-Koehler-Allee 103, 79110 Freiburg, Germany

[3] Institute for Surgical Pathology, Faculty of Medicine, Medical Center – University of Freiburg, Breisacher Strasse 115a, 79106 Freiburg, Germany

[4] Faculty of Biology, University of Freiburg, Schänzlestrasse 1, 79104 Freiburg, Germany

Figure S1. Distribution of different LCM platform across publications from the last decade

Figure S2. Scan of the processed murine kidney before and after LCM

Figure S3. The physical components used to perform the MR-SP^2 workflow

Figure S4. Individual LC-MS/MS runs showing peptide and protein identifications

Figure S5. CAD model showing the surface area for MR-SP^2 and conventional workflow

Figure S6. LC-MS/MS runs of empty Evotips

Figure S7. Heatmap of Pearson's correlation across all samples

Table S1. Vendor-specific keywords used in the literature survey

Table S2. diaPASEF acquisition scheme

Provided as additional supplemental files:

MR-SP^2.mov. Supplemental video showing the workflow steps to perform MR-SP^2

CAD Model Holder.zip. STEP file containing the holder for the microreactor for 3D printing

MR cap.zip. STEP file containing the cap for the microreactor for replication

Supporting Information

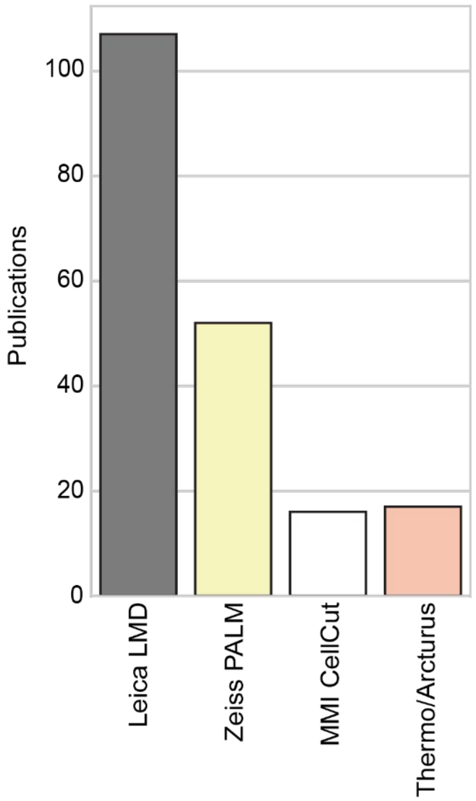

Supplemental Figure 1. *Distribution of different LCM platforms across publications from the last decade. Vendor specific keywords were searched within the Title, Abstract or Material and Methods sections from publications including LCM-coupled to LC-MS/MS available via PubMed API.*

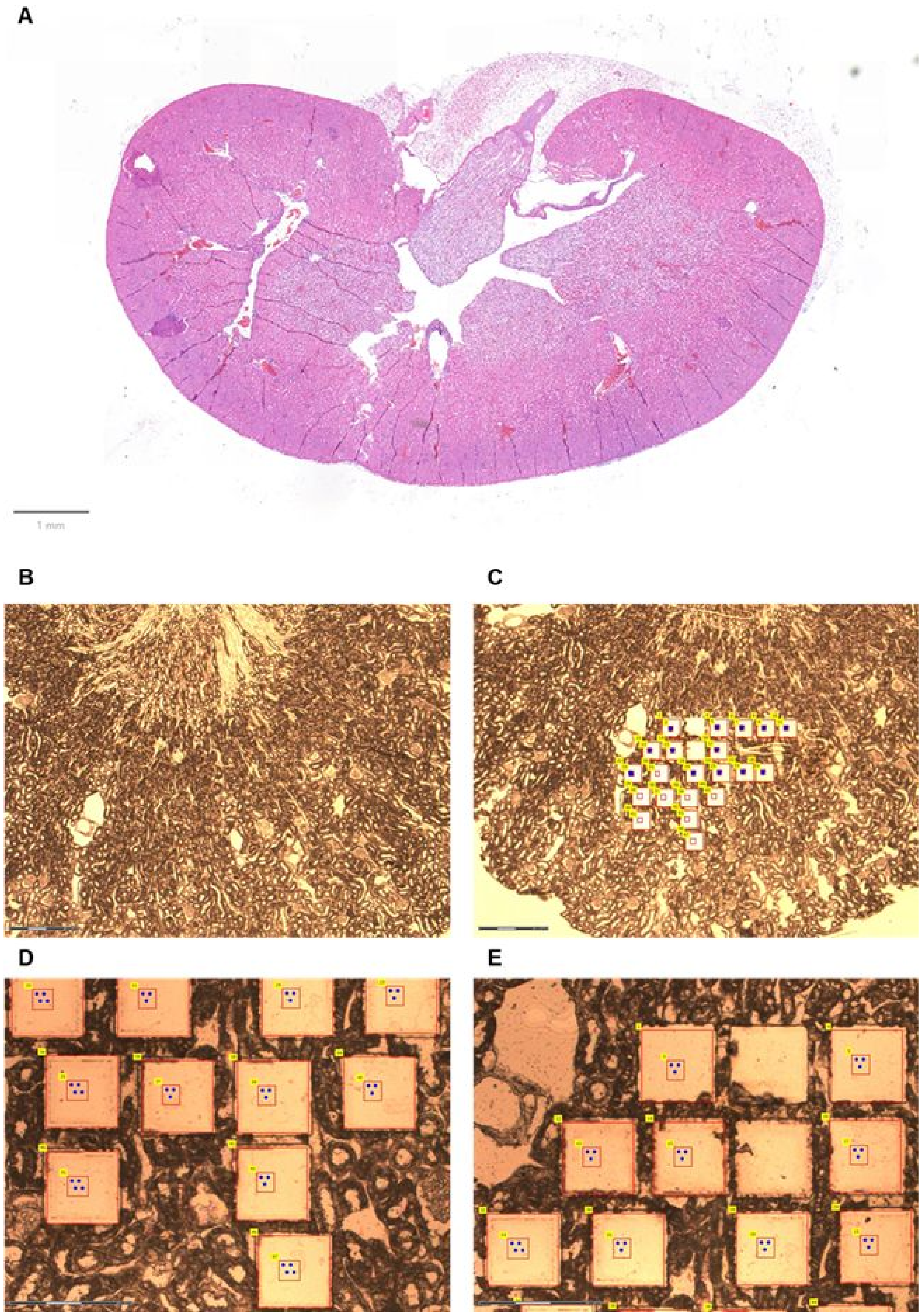

Supplemental Figure 2. (A) *High resolution scan of the murine kidney section after hematoxylin-eosin staining. The scale bar represents 1mm. Microscopic image of the dissected region from deparaffinized murine kidney before* ***(B)*** *and after* ***(C)*** *laser microdissection. The scale bar*

*represents 300 µm. Laser microdissected regions in 20-fold magnification **(D)** and **(E)** with the scale bar representing 150 µm.*

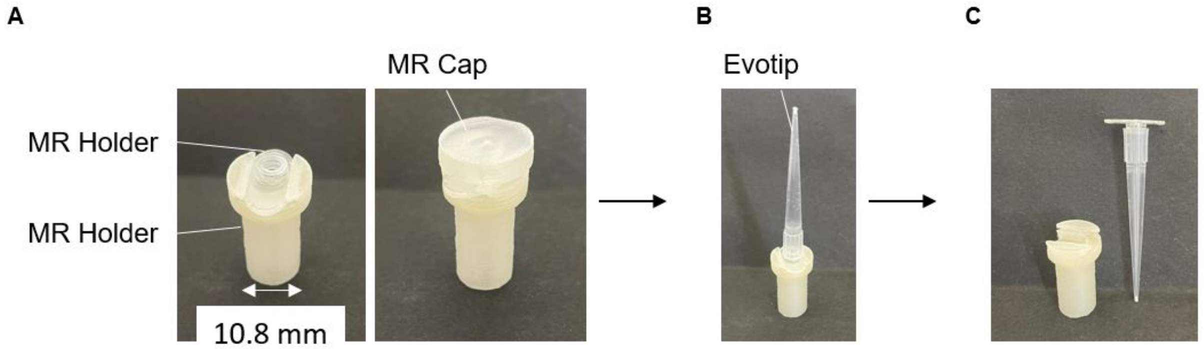

Supplemental Figure 3. (A) *The picture on the left side shows the microreactor placed in the holder for sample processing. The picture on the right side shows the applied microreactor cap when the microreactor is closed.* ***(B)*** *The picture shows the microreactor still in the holder and an Evotip is clipped onto the microreactor.* ***(C)*** *The picture shows the holder and the microreactor clipped on the Evotip after sliding the microreactor and Evotip out of the holder for subsequent centrifugal transfer of the sample into the Evotip.*

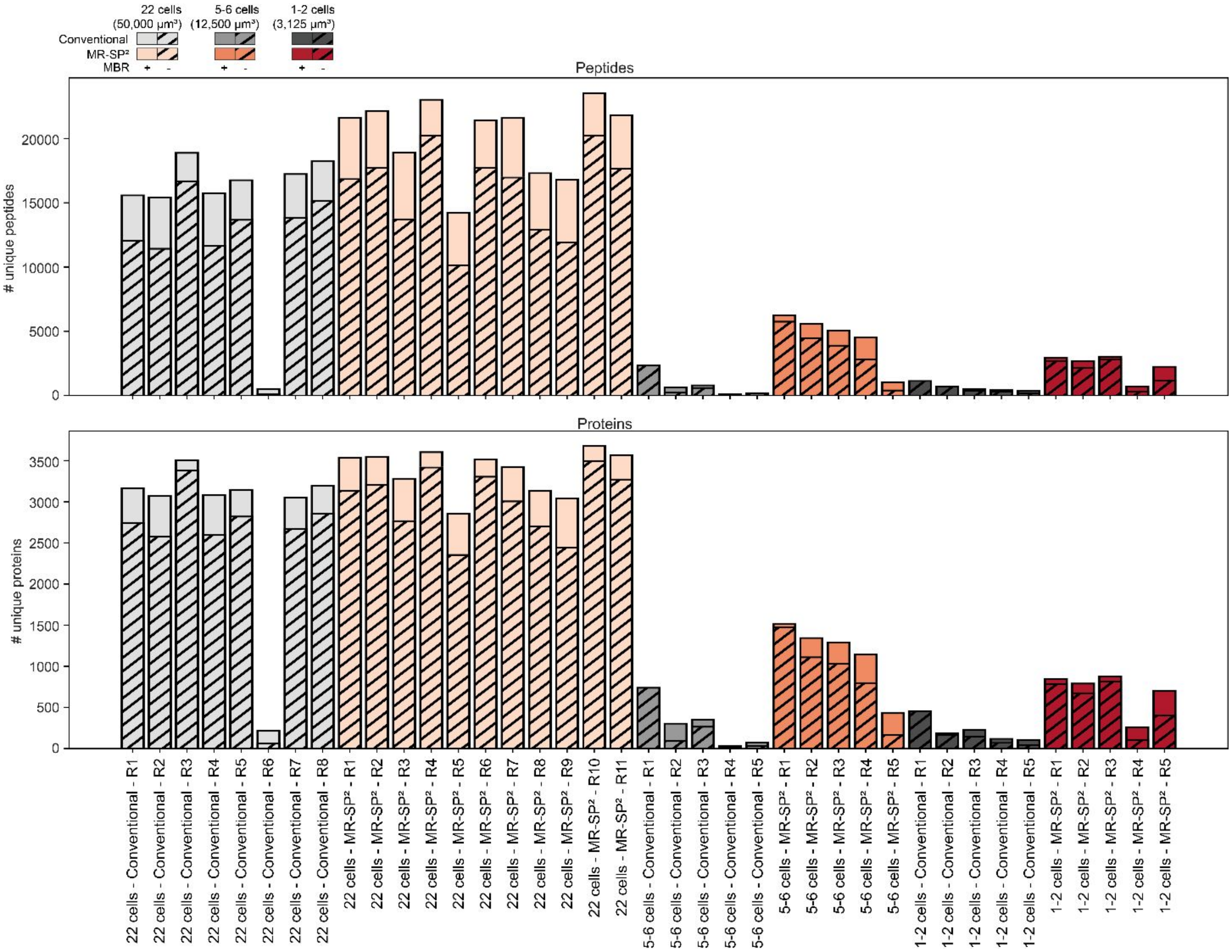

Supplemental Figure 4. *Number of identified peptides and proteins from 22, 5-6 and 1-2 cells of a formalin-fixed murine kidney slide for individual samples. Saturated bars represent identifications without MBR activated, while lighter bars indicate additional identifications attributed to MBR across samples from the same condition.*

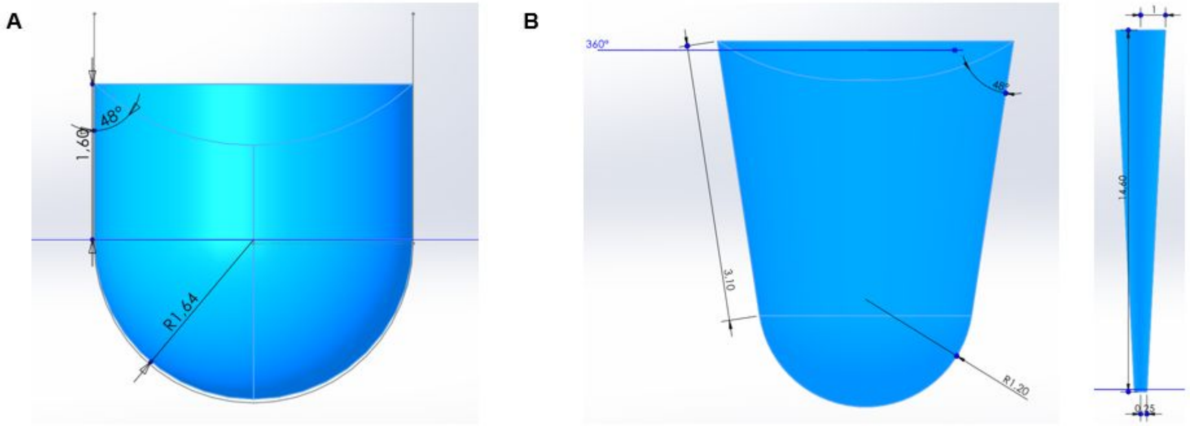

Supplemental Figure 5. (A) *Depicted is the CAD model of the 20 µL sample volume after complete sample processing before transfer to the Evotip. The model shows the exposed polymer surface area to the sample that is responsible for adsorptive losses. The surface area is 33.4 mm². All measurements shown are in mm.* ***(B)*** *Depicted is the CAD model of the 20 µL sample volume after complete sample processing before the transfer to the Evotip (left) in a 200 µL microtube. Additionally, the model on the right shows the 20 µL sample volume in a 100 µL pipette tip that is necessary for transfer to the Evotip. The cumulated surface area of sample processing and pipetting transfer to the Evotip is 92.8 mm². All measurements shown are in mm.*

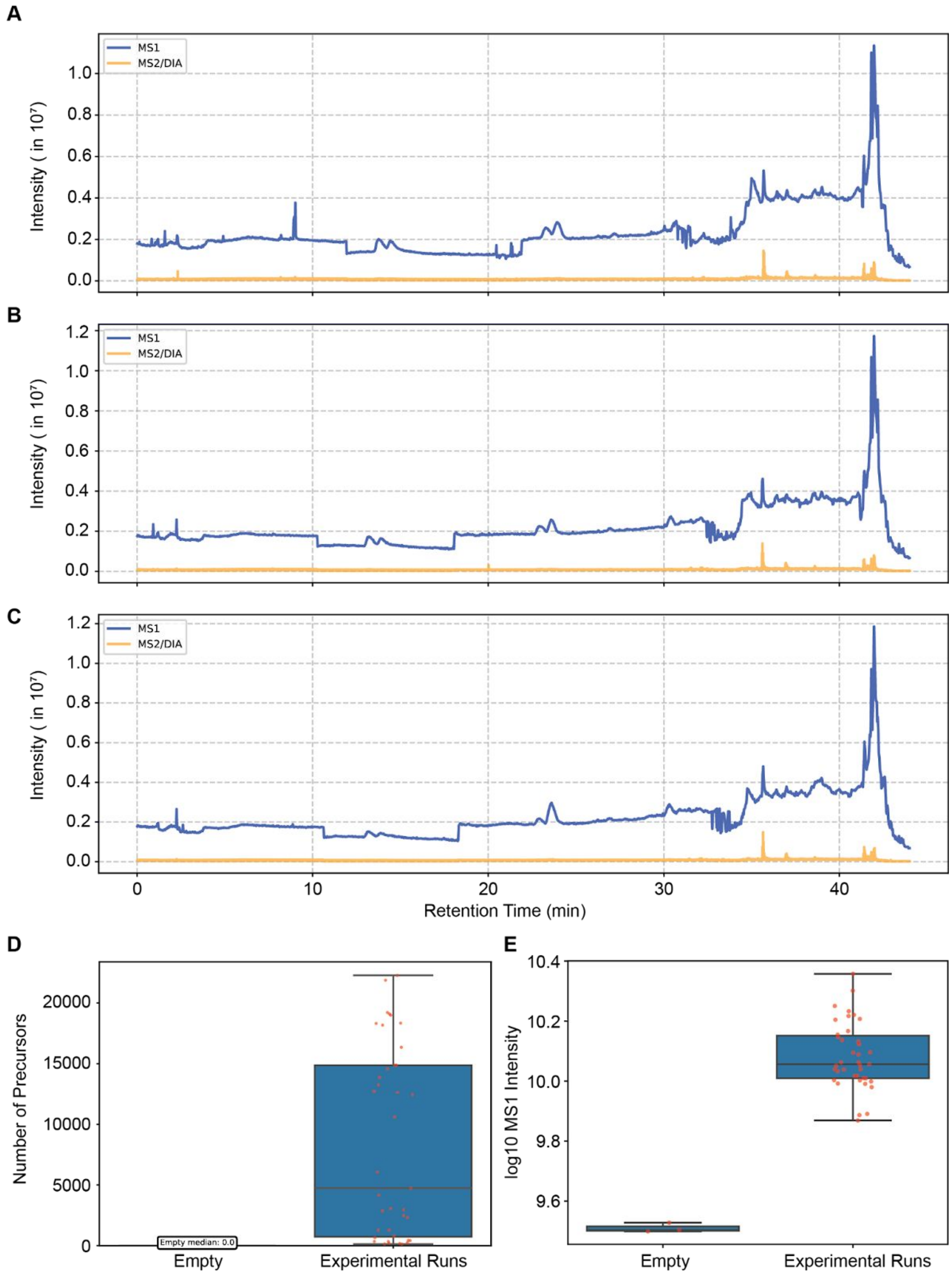

Supplemental Figure 6: *(A-C) Total ion chromatogram from three individual empty runs. (D) Number of identified precursors from empty Evotips or the experimental runs for different cut*

sizes and workflows. (E) Cumulative Log10 intensity of all MS1 scans for the empty runs and the experimental runs. (Empty: n=3; Experimental runs: n=39).

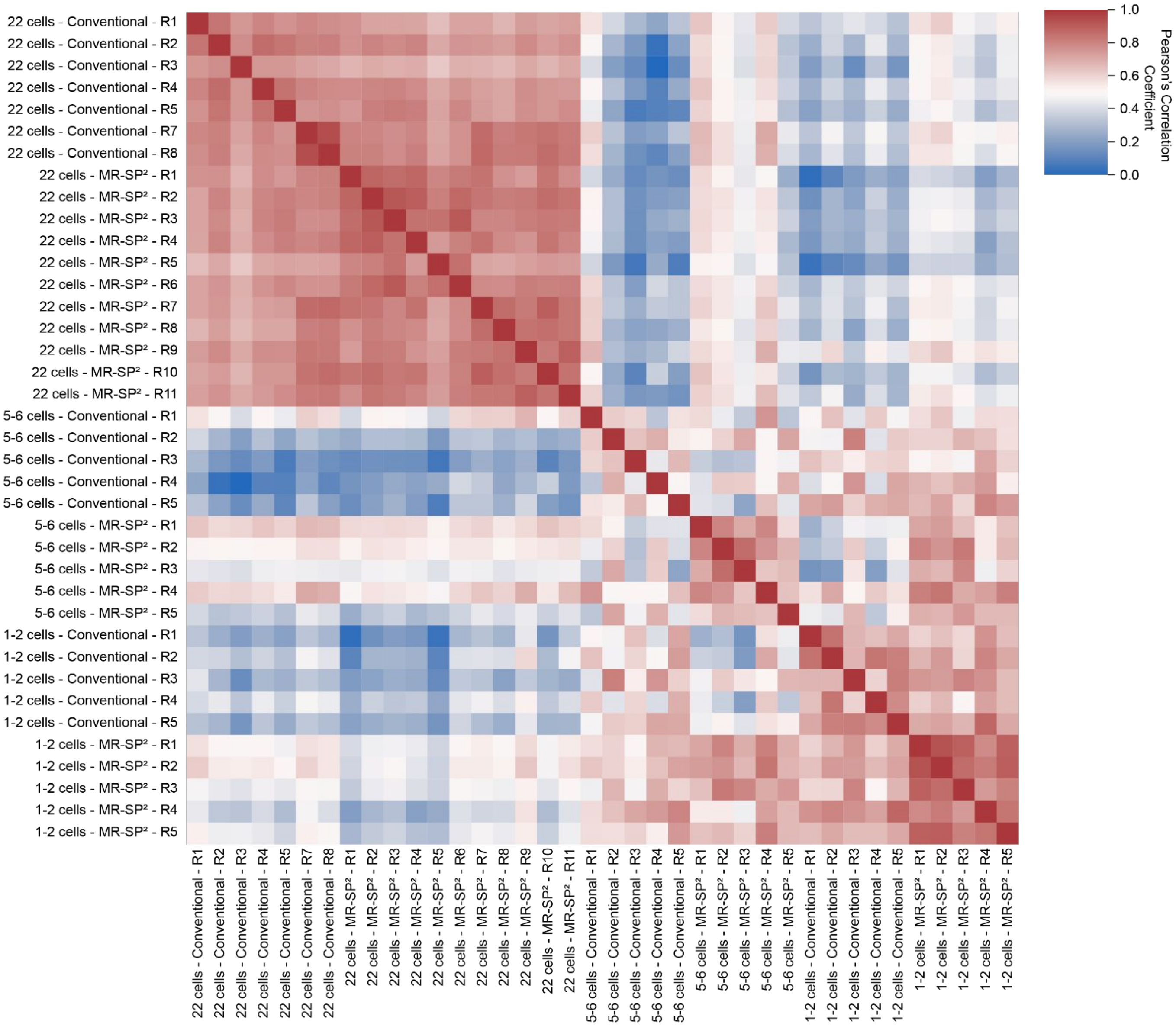

Supplemental Figure 7. *Heatmap of Pearson's correlation coefficient between all samples across all precursors found in >2 samples. High R-values in red indicate good correlation of shared peptide intensity between the two respective samples.*

Supplemental Table 1. *Vendor-specific keywords used in the literature survey.*

Vendor	***Keyword***
ZEISS PALM	*palm microbeam*
ZEISS PALM	*palm micro beam*
ZEISS PALM	*palmmicrobeam*
ZEISS PALM	*zeiss palm*
ZEISS PALM	*palm laser microdissection*
ZEISS PALM	*palm microdissection*
Leica LMD	*leica lmd*
Leica LMD	*lmd7000*
Leica LMD	*lmd7*
Leica LMD	*lmd6*

Leica LMD	*leica microsystems*
MMI CellCut	*mmi cellcut*
MMI CellCut	*mmi cell cut*
MMI CellCut	*molecular machines and industries*
MMI CellCut	*caplift*
Thermo/Arcturus	*arcturusxt*
Thermo/Arcturus	*arcturus xt*
Thermo/Arcturus	*pixcell*
Thermo/Arcturus	*veritas*
Thermo/Arcturus	*arcturus*

Supplemental Table 2. *diaPASEF acquisition scheme.*

#MS Type	Cycle Id	Start IM [1/K0]	End IM [1/K0]	Start Mass [m/z]	End Mass [m/z]	CE [eV]
MS1	0	-	-	-	-	-
PASEF	1	0.6	0.904	400	426	-
PASEF	1	0.91	1.6	800	826	-
PASEF	2	0.6	0.9174	425	451	-
PASEF	2	0.9236	1.6	825	851	-
PASEF	3	0.6	0.9308	450	476	-
PASEF	3	0.9372	1.6	850	876	-
PASEF	4	0.6	0.9442	475	501	-
PASEF	4	0.9507	1.6	875	901	-
PASEF	5	0.6	0.9576	500	526	-

PASEF	5	0.9643	1.6	900	926	-
PASEF	6	0.6	0.971	525	551	-
PASEF	6	0.9779	1.6	925	951	-
PASEF	7	0.6	0.9844	550	576	-
PASEF	7	0.9915	1.6	950	976	-
PASEF	8	0.6	0.9979	575	601	-
PASEF	8	1.0051	1.6	975	1001	-
PASEF	9	0.6	1.0113	600	626	-
PASEF	9	1.0186	1.6	1000	1026	-
PASEF	10	0.6	1.0247	625	651	-
PASEF	10	1.0322	1.6	1025	1051	-
PASEF	11	0.6	1.0381	650	676	-
PASEF	11	1.0458	1.6	1050	1076	-

PASEF	12	0.6	1.0515	675	701	-
PASEF	12	1.0594	1.6	1075	1101	-
PASEF	13	0.6	1.0649	700	726	-
PASEF	13	1.073	1.6	1100	1126	-
PASEF	14	0.6	1.0783	725	751	-
PASEF	14	1.0865	1.6	1125	1151	-
PASEF	15	0.6	1.0918	750	776	-
PASEF	15	1.1001	1.6	1150	1176	-
PASEF	16	0.6	1.1052	775	801	-
PASEF	16	1.1137	1.6	1175	1201	-
